# Validity and Reliability of an Inertial Device for Measuring Dynamic Weight-Bearing Ankle Dorsiflexion

**DOI:** 10.3390/s20020399

**Published:** 2020-01-10

**Authors:** José M. Oliva-Lozano, Isabel Martín-Fuentes, José M. Muyor

**Affiliations:** 1 Health Research Centre; Faculty of Educational Sciences, University of Almería, 04102 Almería, Spain; jol908@ual.es (J.M.O.-L.); imf902@ual.es (I.M.-F.); 2 Laboratory of Kinesiology, Biomechanics and Ergonomics, Research Central Services, University of Almería, 04102 Almería, Spain

**Keywords:** inertial sensors, wearable sensors, kinematics, WIMU

## Abstract

A decrease in ankle dorsiflexion causes changes in biomechanics, and different instruments have been used for ankle dorsiflexion testing under static conditions. Consequently, the industry of inertial sensors has developed easy-to-use devices, which measure dynamic ankle dorsiflexion and provide additional parameters such as velocity, acceleration, or movement deviation. Therefore, the aims of this study were to analyze the concurrent validity and test-retest reliability of an inertial device for measuring dynamic weight-bearing ankle dorsiflexion. Sixteen participants were tested using an inertial device (WIMU) and a digital inclinometer. Ankle dorsiflexion from left and right ankle repetitions was used for validity analysis, whereas test-retest reliability was analyzed by comparing measurements from the first and second days. The standard error of the measurement (SEM) between the instruments was very low for both ankle measurements (SEM < 0.6°). No significant differences between instruments were found for the left ankle measurement (*p* > 0.05) even though a significant systematic bias (~1.77°) was found for the right ankle (*d* = 0.79). R^2^ was very close to 1 in the left and right ankles (R^2^ = 0.85–0.89) as well as the intraclass correlation coefficient (ICC > 0.95). Test-retest reliability analysis showed that systematic bias was below 1° for both instruments, even though a systematic bias (~1.50°) with small effect size was found in the right ankle (*d* = 0.49) with WIMU. The ICC was very close to 1 and the coefficient of variation (CV) was lower than 4% in both instruments. Thus, WIMU is a valid and reliable inertial device for measuring dynamic weight-bearing ankle dorsiflexion.

## 1. Introduction

Ankle dorsiflexion is defined as the movement that decreases the angle between the foot and the leg, in which the shin is brought towards the toes. Considering that the gastrocnemius and soleus muscles are plantar flexors, a decrease in ankle dorsiflexion range of motion is observed when these muscles are shortened, which causes a change in biomechanics as a result of ankle pronation and knee flexion [1].

In addition, ankle dorsiflexion is considered a crucial component of lower limb balance and flexibility [2]. For example, restricted ankle flexibility could alter jumping kinematics by increasing landing forces, which predispose the athlete to get injured [3]. In this sense, from a clinical perspective, the evaluation of the ankle dorsiflexion is important in terms of lower limb injury prevention [3]. A reduced ankle dorsiflexion is associated with the risk of suffering anterior cruciate ligament injury [4,5], plantar fasciitis [6], Osgood Schlatter disease [7], and patellar tendinopathy [8,9].

A weight-bearing lunge test is considered the most representative measure of ankle dorsiflexion since it provides consistent results when tested by one or more practitioners [10]. This is particularly important in team sports and research contexts where participants need to be continually reevaluated. Currently, there are different instruments that could be applied to weight-bearing lunge test, which include traditional goniometers [11], digital inclinometers [12,13], and smartphones [13,14]. These instruments measure weight-bearing lunge test with static conditions (not dynamic) and variables such as velocity (degrees per second), acceleration, or deviation cannot be obtained unless optical tracking systems [5,15], stretch sensors [16], electrogoniometers [17], or inertial measurement units [18,19] are used. Although most of these instruments may accurately measure ankle dorsiflexion [5,11,12,13,14,17], there are some disadvantages. Goniometers and digital inclinometers, which have good portability and ease of use, depend on the tester’s reliability [11,12]. In addition, optical tracking systems, which are the gold standard for motion analysis, require a complex installation and data analysis [19]. Furthermore, instruments such as smartphones, stretch sensors, electrogoniometers, and inertial sensors, which are cheaper than optical tracking systems and provide similar variables, need accurate calibration procedures that may affect measurement accuracy [17,19].

Since most human physical activities are performed dynamically, such instruments need to be able to measure angular displacement under dynamic conditions in order to be considered an effective tool [20]. For example, specific training of ankle dorsiflexion velocity and acceleration may have validity for the study of gait-related interventions by improving temporal symmetries during stance and swing [21]. Then, the knowledge of these variables could provide an insight into changes in motor control for walking or running [21]. Therefore, the industry of inertial sensors has developed devices which can measure human body segment orientation when integrating acceleration and angular velocity signals [22]. The main limitation of inertial sensors is related to the errors of drift and distortion [18]. Given these errors that occur when using inertial sensors, a multi-sensor fusion from 3D accelerometers, gyroscopes, and magnetometers is considered necessary because the combination of data from different sensors may provide more accurate data [23]. For example, the accelerometers may compensate for the drift of the gyroscopes on horizontal axes whereas magnetometers solve the drift on the vertical axis [24]. Currently, there are several wearable sensors available on the market for research purposes and clinical applications [19]. The rapid growth of inertial sensors technology has led to challenges regarding the design, research, and development work for human activity monitoring [16,25,26]. However, improvements in data logging, data processing, and device attachment, as well as validation studies of inertial sensors, should be made in order to use these systems more widely [18,24].

Hence, the aims of this study were: (1) to analyze the concurrent validity of an inertial device for measuring dynamic weight-bearing ankle dorsiflexion; (2) to analyze the test-retest reliability of an inertial device for measuring dynamic weight-bearing ankle dorsiflexion.

## 2. Materials and Methods

### 2.1. Participants

Sixteen professional soccer players (age: 26.33 ± 4.10 years old; height: 179.22 ± 5.66 cm; weight: 75.56 ± 7.36 kg) from a team competing in *LaLiga* were evaluated. The sample size was estimated a priori with a statistical power above 0.8 and was of similar size to previous studies [13,27]. All participants were informed of the nature of the study and signed informed consent. As exclusion criteria, if any participant reported pain or injury during the last two months in the ankle, gastrocnemius, or soleus muscles, the participant would be excluded from the test.

The research team was given authorization from the club to carry out the study within the team’s facilities. After providing written informed consent, the test was explained to the participants one by one.

### 2.2. Weight-Bearing Lunge Test

This test was used in order to measure dynamic ankle dorsiflexion range of motion from the right and left leg. There is strong evidence that a weight-bearing lunge test is recommended for ankle dorsiflexion assessment since good inter-clinician and intra-clinician reliability is provided (minimum detectable change: 4.6° and 4.7°, respectively) [10]. Participants were instructed to move into a weight-bearing lunge position, in which a foot stands in the front with a 90 degree knee and ankle flexion, whereas the back foot is in plantar flexion with the knee on the ground. Then, they lunged forward and the range of motion was measured.

The experiment consisted of a total of two repetitions (one for the left ankle and one for the right ankle) with a two-minute break between repetitions. The experiment was repeated two weeks later at the same time of day [28]. The range of motion of the left and right ankle repetitions was used for validity analysis, whereas test-retest reliability was analyzed by comparing the range of motion from the first to the second experiment of each ankle. In addition, participants were measured (without any type of warm-up) one by one by the same testers. One tester checked that the technique was correct, paying special attention to the front foot full heel contact with the ground. Another tester placed the instruments and a third collected the data.

### 2.3. Instruments

**Figure 1:**
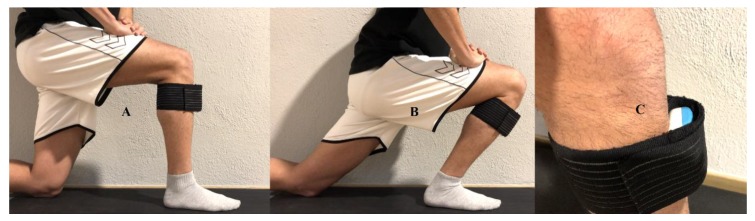
Athlete wearing a WIMU Pro device for the measurement of weight-bearing ankle dorsiflexion. (**A**) starting point of weight-bearing lunge test; (**B**) ending point of weight-bearing lunge test; (**C**) WIMU Pro device in touch with the tibia.

An inertial device, the WIMU Pro system (RealTrack Systems, Almería, Spain), was used for the dynamic measurement of ankle dorsiflexion (Figure 1). It consists of various inertial sensors (four 3D accelerometers, three 3D gyroscopes, one magnetometer, one barometer) which collected data at a 1000 Hz sampling rate. The device was placed in a vertical position on the proximal tibia by aligning the Z-axis of the device with the tibia. The back of the device (white side) was in touch with the tibia and an elastic band (Aptonia, Lille, France) fixed the device. The device was calibrated right before the start of the test following the manufacturer’s instructions on WIMUNET (RealTrack Systems, Almería, Spain). Then, the device was placed in a steady surface. The device was turned on and 30 s were left until the session began to be recorded.

**Figure 2:**
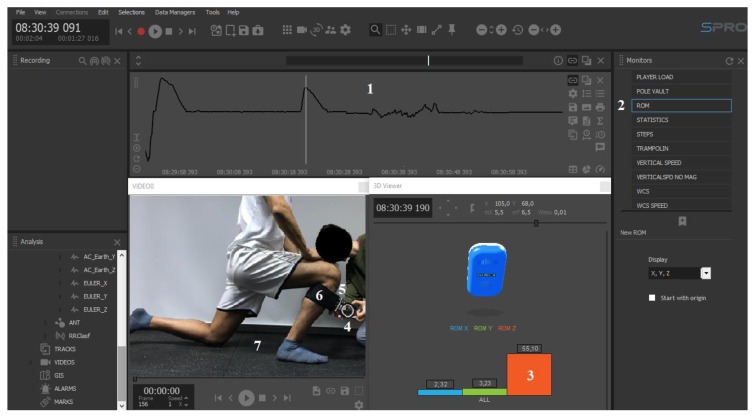
Description of SPro software interface. 1: raw data from the Euler Z channel collected by WIMU Pro; 2: ROM application available on Monitors panel; 3: range of motion registered in ankle dorsiflexion movement; 4: inclinometer; 5: WIMU Pro under the inclinometer; 6: elastic fixing band; 7: video recording of the experimental setting synchronized with the data.

The WIMU Pro system works with a multi-sensor fusion and transfers data to the SPro software (RealTrack Systems, Almería, Spain), which provides raw data from the “Euler Z channel”. Specifically, range of motion was calculated by using the “ROM monitor” of SPro software (Figure 2), which integrates different algorithms that report live data wirelessly from the device to SPro through the NanoStation M5 (Ubiquiti Inc., New York, NY, USA).

The data from the inertial device was compared to a Unilever inclinometer (ISOMED, Inc., Portland, OR, USA). The inclinometer was considered as the reference instrument because it is defined as a valid and reliable instrument for assessing ankle dorsiflexion [14,29,30] and several other joints’ range of motion (e.g., hip flexion) [31]. The inclinometer was placed on top of the device following similar previous testing procedures [31] and the start point was set at a 90 degree knee flexion.

### 2.4. Statistical Analysis

First, a Shapiro–Wilk normality test was conducted in order to analyze normality of variables. Since the variables were normally distributed, paired students’ *t*-tests were used to compare the data collected by both instruments (concurrent validity) and both repetitions (test-retest reliability). Effect sizes for between-groups effects (Cohen’s *d*) were calculated by a combined standard deviation and categorized as: trivial (0–0.19), small (0.20–0.49), medium (0.50–0.79), and large (≥0.8) [32].

The concurrent validity of WIMU Pro was analyzed by calculating the difference between instruments (systematic bias), least squares linear regression [33], standard error of measurement (SEM), and intraclass correlation coefficient (ICC) (2,1) with 95% confidence intervals (CI). For the test-retest reliability of WIMU Pro, ICC (2,1) with 95% CI and coefficient of variation (CV) percentage were also measured.

The statistical power and Cohen’s *d* was calculated with G*Power 3.1 [34], and the rest of the statistical analysis with IBM SPSS Statistics (IBM Corp., Armonk, NY, USA). The statistical power was > 0.9 for all variables analyzed with the sample size used in the present study. The level of significance was set at *p* ≤ 0.05.

## 3. Results

Table 1 shows descriptive statistics of ankle dorsiflexion measured by the reference instrument (inclinometer) and WIMU Pro. The standard error of the measurement (SEM) was very low for both ankle measurements (SEM < 0.6°). There were no significant differences between instruments for left ankle measurement (*p* > 0.05) even though a significant systematic bias (~1.77°) with medium effect size was found for the right ankle (*d* = 0.79). However, R^2^ was very close to 1 in the left ankle (R^2^ = 0.85) and the right ankle (R^2^ = 0.89) as well as the ICC 1 in both ankles (ICC > 0.95). 

**Figure 3:**
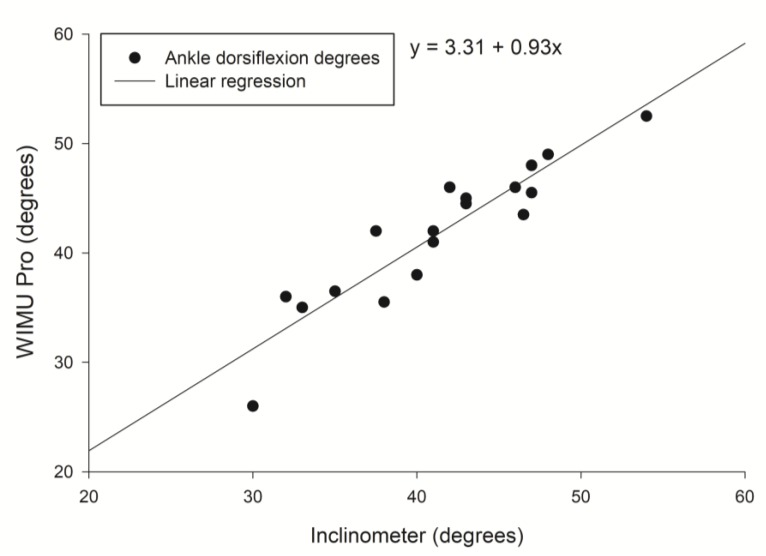
Correlation between left ankle dorsiflexion (degrees) measured by the inclinometer and WIMU Pro.

**Figure 4:**
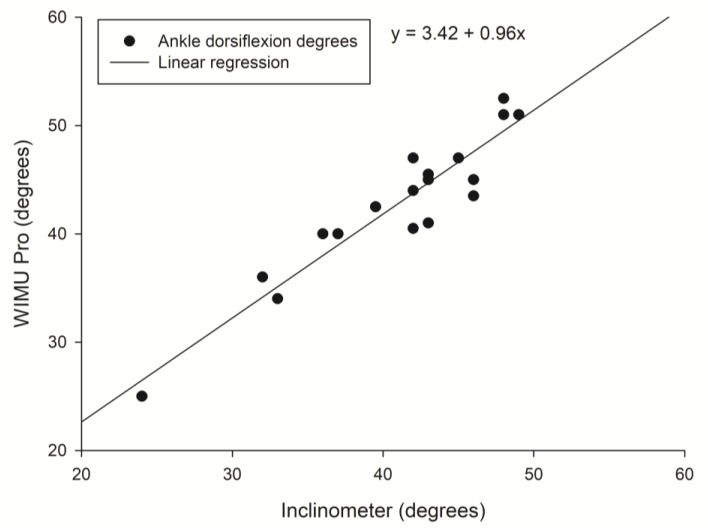
Correlation between right ankle dorsiflexion (degrees) measured by the inclinometer and WIMU Pro.

Correlation between ankle dorsiflexion degrees was evaluated with the inclinometer and the WIMU Pro system. The solid line shows the linear regression fit of the 2 systems with the associated regression equation. Data points represent individual dorsiflexion degrees for left ankle (Figure 3) and right ankle (Figure 4).

Table 2 shows very high test-retest reliability results for both instruments. The systematic bias was lower than 1° for both instruments even though a systematic bias (~1.50°) with small effect size was found in right ankle (*d* = 0.49) with WIMU Pro. However, SEM was higher for the inclinometer (~0.80°) than WIMU Pro (~0.52°). In addition, ICC was very close to 1 and CV was lower than 4% in both instruments.

## 4. Discussion

The purpose of this study was to analyze the concurrent validity and test-retest reliability of a novel inertial device for measuring dynamic weight-bearing ankle dorsiflexion. The main findings were that WIMU Pro was a valid and reliable device for measuring dynamic weight-bearing ankle dorsiflexion.

WIMU Pro could be considered as a valid instrument since data showed a very good association between the reference instrument (inclinometer) and the inertial device. SEM was very low (SEM < 0.6°). There were no significant differences between instruments (*p* > 0.05) when measuring left ankle dorsiflexion, and a significant systematic bias (~1.77°) with a medium effect size being found for the right ankle (*d* = 0.79), which was below the accuracy of the reference instrument (±2°) [31]. In addition, this inertial device has been previously validated for clinical purposes (e.g., hamstring extensibility in the passive straight leg raise test [31]). This study showed similar results in systematic bias (<0.5°), SEM (<0.43°), and an ICC very close to 1 (0.99) [31]. However, there are studies that have specifically validated other instruments for measuring weight-bearing ankle dorsiflexion and the following SEMs were found: 0.48° when testing a smartphone app [13], 2.09° in an achillometer [35], and 6.99° with an electrogoniometer [17]. Systematic differences were also found: 0.54° [13] and 0.77° [36]. The results from this study were also in line with the following statistics reported: ICC = 0.71 [35], ICC = 0.83 [14], ICC = 0.97 [37], R^2^ = 0.93 [17], and R^2^ = 0.99 [13].

Additionally, the test-retest reliability analysis was conducted in order to confirm that the observed differences when measuring ankle dorsiflexion were not due to systematic errors of measurement or random errors caused by mechanical variations [38]. WIMU Pro showed a very good test-retest reliability since the systematic bias was lower than the accuracy of the reference instrument (± 2°) [31] and SEM was lower (~0.52°) than SEM in the inclinometer (~0.80°). Indeed, the maximum CV reported by WIMU Pro was 3.08% (CVs below 10% are considered acceptable for analytic purposes) [38]. The test-retest reliability of WIMU Pro was analyzed in a previous study which reported ICCs very close to 1 (0.972) as well as very low SEM (0.31°) and CV (0.01%) when measuring hip flexion range of motion [31]. Moreover, the following test-retest SEMs, when measuring ankle dorsiflexion with other devices in previous studies, were greater than in the current study: 0.43° [36], 1.40° [35], 2.4° [35,37], and 3.63° [17]. Moreover, ICCs obtained with WIMU Pro in the current study were similar or higher than in other studies that evaluated ankle dorsiflexion with other devices: 0.85 [35], 0.94 [39], and 0.97 [13,17,36].

However, this study has some limitations. On the one hand, the participants were active athletes without any injury, so the mechanics of the ankle dorsiflexion movement had no alterations. On the other hand, the measurements were limited to ankle dorsiflexion and other ankle movements such as: plantar flexion, inversion, or eversion, and measuring all ankle movements during different types of tasks such as: squatting, jumping, walking, running, etc. have not yet been tested. In addition, the inter-device validity and reliability were not analyzed. Future studies are needed to test the validity and reliability of WIMU Pro in other populations as well as the inter-device validity and reliability. Additionally, these studies may be carried out using optical tracking systems as a gold-standard instrument.

The potential practical application of this study is that WIMU Pro accurately registers dynamic ankle dorsiflexion measures, which may also be synchronized with numerous variables obtained by this device (e.g., velocity, acceleration, balance). The use of wearable sensors is considered necessary for human activity monitoring when it comes to maximizing athletic performance, injury prevention, or monitoring physical activity in clinical, pathological, and aging populations [16,25]. WIMU Pro could serve as a multi-purpose instrument for human activity monitoring since it now has an additional function to the ones provided by it, which have already been investigated. These include, for example, hamstring extensibility [31] and gait analysis [40], strength and conditioning training (e.g., velocity in resistance exercises, countermovement jump) [41,42,43], neuromuscular running load monitoring [44], and other physical monitoring in general [45].

## 5. Conclusions

WIMU Pro is a valid and reliable inertial device for measuring dynamic weight-bearing ankle dorsiflexion. This instrument could be used to analyze the kinematic parameters derived from ankle dorsiflexion range of motion.

## Figures and Tables

**Table 1:** Concurrent validity analysis for measuring ankle dorsiflexion.

	Left Ankle	Right Ankle
Inclinometer (°; 95% CI)	41.33 ± 6.30 (38.19–44.47)	41.02 ± 6.50 (37.79–44.26)
WIMU Pro (°; 95% CI)	41.77 ± 6.35 (38.61–44.93)	42.80 ± 6.63 (39.50–46.10)
Systematic bias (°)	−0.44 ± 2.47	−1.77 ± 2.23 *
Cohen’s *d*	0.17	0.79
SEM (°)	0.58	0.52
R^2^ correlation	0.853 †	0.888 †
ICC (95% CI)	0.961 † (0.898–0.985)	0.954 † (0.766–0.986)

°: degrees; mean ±: standard deviation; CI: confidence interval; SEM: standard error of the measurement; ICC: intraclass correlation coefficient; * *p* ≤ 0.01; † *p* ≤ 0.001.

**Table 2:** Test-retest reliability analysis for measuring ankle dorsiflexion.

	Inclinometer		WIMU Pro	
	Left Ankle	Right Ankle	Left Ankle	Right Ankle
Test (°; 95% CI)	40.83 ± 6.52 (37.58–44.08)	40.61 ± 6.64 (37.30–43.91)	41.39 ± 6.42 (38.19–44.58)	42.06 ± 6.53 (38.80–45.30)
Retest (°; 95% CI)	41.83 ± 6.32 (38.68–44.98)	41.44 ± 6.81 (38.05–44.83)	42.17 ± 6.40 (38.98–45.35)	43.56 ± 6.91 (40.11–46.99)
Systematic bias (°)	−1.00 ± 2.47	−0.83 ± 3.43	−0.77 ± 1.73	−1.50 ± 2.22 *
Cohen’s *d*	0.34	0.27	0.27	0.49
SEM (°)	0.58	0.80	0.40	0.52
ICC (95% CI)	0.958 † (0.884–0.984)	0.930 † (0.817–0.974)	0.979 † (0.939–0.992)	0.961 † (0.845–0.987)
CV (%)	3.06	3.75	2.24	3.08

°: degrees; mean ± standard deviation; CI: confidence interval; SEM: standard error of the measurement; ICC: intraclass correlation coefficient; CV: coefficient of variation; * *p* ≤ 0.01; † *p* ≤ 0.001.
